# Novel Nucleic Acid Binding Small Molecules Discovered Using DNA-Encoded Chemistry

**DOI:** 10.3390/molecules24102026

**Published:** 2019-05-27

**Authors:** Alexander Litovchick, Xia Tian, Michael I. Monteiro, Kaitlyn M. Kennedy, Marie-Aude Guié, Paolo Centrella, Ying Zhang, Matthew A. Clark, Anthony D. Keefe

**Affiliations:** 1 X-Chem Pharmaceuticals, Waltham, MA 02435, USA; alitovchick@x-chemrx.com (A.L.); mmonteiro@x-chemrx.com (M.I.M.); Kaitlyn.kennedy1288@gmail.com (K.M.K.); mguie@x-chemrx.com (M.-A.G.); pcentrella@x-chemrx.com (P.C.); yzhang@x-chemrx.com (Y.Z.); mclark@x-chemrx.com (M.A.C.); 2 Arrakis Therapeutics, Waltham, MA 02451, USA; xia2tian@hotmail.com

**Keywords:** DNA-encoded chemical library, affinity-mediated selection, G-quartet, c-myc, SPR

## Abstract

Inspired by the many reported successful applications of DNA-encoded chemical libraries in drug discovery projects with protein targets, we decided to apply this platform to nucleic acid targets. We used a 120-billion-compound set of 33 distinct DNA-encoded chemical libraries and affinity-mediated selection to discover binders to a panel of DNA targets. Here, we report the successful discovery of small molecules that specifically interacted with DNA G-quartets, which are stable structural motifs found in G-rich regions of genomic DNA, including in the promoter regions of oncogenes. For this study, we chose the G-quartet sequence found in the *c-myc* promoter as a primary target. Compounds enriched using affinity-mediated selection against this target demonstrated high-affinity binding and high specificity over DNA sequences not containing G-quartet motifs. These compounds demonstrated a moderate ability to discriminate between different G-quartet motifs and also demonstrated activity in a cell-based assay, suggesting direct target engagement in the cell. DNA-encoded chemical libraries and affinity-mediated selection are uniquely suited to discover binders to targets that have no inherent activity outside of a cellular context, and they may also be of utility in other nucleic acid structural motifs.

## 1. Introduction

DNA-encoded chemical libraries have established a reputation as a source of novel small molecule hits for a variety of therapeutically relevant protein targets [1]. Most reported projects have utilized DNA-encoded chemical libraries that were prepared using DNA-recorded chemistry, in which successive “split and pool” steps are utilized with DNA sequence variation encoding the chemical steps: However, other methodologies also exist [1,2]. Hits are discovered by the deep-sequencing of the encoding DNA contained in selection outputs, permitting the identification of library members that have been enriched as a result of affinity-mediated selection by retaining binding to the target through wash steps. Affinity-mediated selection is uniquely suited to discover target-interacting small molecules that have no inherent activity outside of a cellular context, as binding events directly mediate enrichment. The ability to rapidly query libraries of 10^8^–10^11^ individual unique compounds in which all library members are mixed together using miniaturized affinity-based selection methods allows for multiple selection experiments with different targets or otherwise varying conditions to be explored in parallel [3]. These conditions can be varied to focus the selection campaign on the desired characteristics of the small-molecule output. Altering the target concentration, the inclusion of selectivity targets, and the addition of known binders to compete with library compounds are individual variables that can be explored in parallel in a single-selection campaign. We have previously reported the result of multiple parallel selection conditions that successfully identified novel small-molecule inhibitors of therapeutic protein targets, including BTK [3], PAR2 [4], sEH [5], InhA [6], Mcl1 [7], and ATAD2 [8]: Other groups have reported successes with similar platforms [1].

Since 2012, we have licensed over 60 small-molecule programs for different protein targets, including kinases, enzymes, protein–protein interaction inhibitors, G-Protein Coupled Receptors (GPCR’s), etc. Encouraged by this success and in light of growing interest in targeting DNA and RNA, we decided to assess the potential for our platform to provide modulators for these classes of targets. The only other example that we are aware of that has utilized DNA-encoded chemical libraries to target a nucleic acid was in a verbal communication at a recent conference [9].

We chose to focus our initial efforts on DNA G-quartets as a target class because of their compact stable structures, which are formed by π-stacked quartets of hydrogen-bonded guanines that provide remarkable conformational stability to short sequences of DNA [10]. Each G-quartet comprises a four-stranded antiparallel structure, within which each guanine interacts with two others through a network of Hoogsteen hydrogen bonds to form planar cyclic arrangements of four guanines. These structures are further stabilized by interactions with monovalent cations, such as potassium or sodium.

One of the most remarkable G-quartet examples is the telomere [11]. Telomeres are DNA sequences that comprise large numbers of guanine-rich tandem repeats at the ends of eukaryotic chromosomes. Telomeres contain large numbers of G-quartet structures that protect against genomic instability by stabilizing their termini. Telomeres are in turn maintained by telomerase, which plays a major role in cellular immortalization by catalyzing telomere extension. In the mid-1990s, it was recognized that telomerase is upregulated in many human cancers, stimulating interest in both telomerase and telomeres as potential anticancer targets [12]. One approach focused on targeting telomere sequences that could form G-quartets using phenotypic screening [12,13]. Telomerase has reverse-transcriptase activity and creates additional 5′-TTAGGG-3′ repeats at the telomere 3′-terminus: Telomeres that adopt a G-quartet structure inhibit the enzyme’s catalytic activity [14].

Another therapeutically relevant example of a G-quartet is found in the promoter region of the *c-myc* gene, which controls the expression of c-myc [15,16,17]. Overexpression of c-myc is associated with a variety of malignant tumors, including Burkitt’s lymphoma [18]. Human c-myc is located on chromosome 8q24.1 [18] and consists of three exons and two introns [18,19]. Transcription of c-myc is regulated by multiple promoters. The nuclease hypersensitivity element III1 (NHEa III1) forms an antiparallel G-quartet structure upstream of the P1 promoter of c-myc that functions as a transcriptional repressor and controls 80–90% of c-myc transcription levels [20,21]. Transcription factors such as nucleolin [22] bind G-quartets, and in particular the c-myc G-quartet, which suggests a potential mechanism of transcription repression. 

Several other oncogenes also have G-quartet regulatory elements in their promoters [15,23,24]. Multiple compounds have been identified as potential c-myc inhibitors [15,24,25], including a compound that entered a Phase I clinical trial [26]. If robust new strategies for specifically targeting gene promoter G-quartet structures could be established, their therapeutic potential could be broad [15]. However, despite considerable efforts, a G-quartet-modulating drug is not yet available, and molecules that discriminate between different G-quartet structures have proven challenging to discover [17].

We used the affinity-mediated selection of a set of DNA-encoded chemical libraries to try to discover specific high-affinity binders to oligonucleotides containing G-quartets. G-quartet target oligonucleotides are easy to synthesize and readily available from many vendors, including in affinity-tagged forms (e.g., biotinylated). Most DNA synthesis vendors can provide tagged DNA oligos of sufficient quantity (hundreds of micrograms) and purity (95–98% through HPLC) for direct use in affinity-mediated selection experiments. The fact that DNA targets are readily chemically synthesized and easily refolded in solution makes them significantly easier to access and handle than protein targets. Here, we demonstrate the affinity-mediated selection of DNA-encoded chemical libraries as a discovery methodology for small-molecule binders of the c-myc promoter and demonstrate that these compounds bind their targets using Surface Plasmon Resonance (SPR), a homogeneous solution-phase PCR-stop assay, and we also indicate target engagement in Burkitt lymphoma cell line RA-1 RAMOS, ATCC^®^ CRL-1596 [19] by observing effects upon the expression of c-myc under the control of a G-quartet-containing promoter.

## 2. Results

### 2.1. DNA-Encoded Chemical Libraries

**Figure 1:**
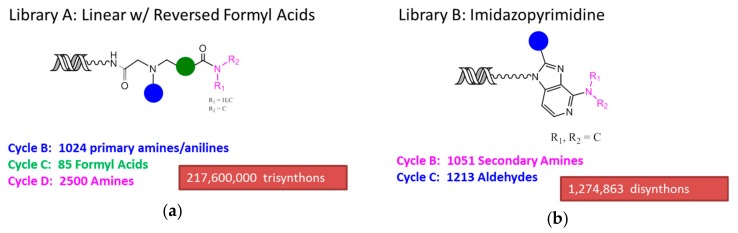
Schemes of the DNA-encoded libraries A and B: (**a**) Library A was a three-cycle linear library with formyl acid cores comprising 217.6 million individual unique compounds; (**b**) Library B was a two-cycle imidazopyrimidine core library comprising 1.275 million individual unique compounds.

We used a combined deck of 33 different DNA-encoded chemical libraries for this study. Compounds derived from two of these libraries, A and B, are reported. Individual DNA-encoded chemical libraries were synthesized using a DNA-recorded split and pool procedure in which DNA tagging and building block installation events occurred during each split. We have described this general methodology before [3,4,5,6,7,8,27,28]. The synthetic schemes defining each library (A and B) are shown in Figure 1. Library A contained 218 million individual unique encoded compounds, each with three diversity points arranged linearly and with connectivity defined by, successively, a reductive alkylation and an acylation. Library B contained 1.3 million individual unique encoded compounds, each with two diversity points displayed on an imidazopyrimidine core. All libraries were synthesized at X-Chem Pharmaceuticals in Waltham, Massachusetts, USA.

### 2.2. Oligonucleotide Synthesis

DNA oligonucleotides were synthesized by Integrated DNA Technologies (Coralville, Iowa, USA) in both biotinylated and nonbiotinylated variants at 1-µmol scale and were HPLC-purified by the vendor. The oligonucleotide sequences used in this study are shown in Table 1 and example LCMS data are shown in Figure S2.

### 2.3. Affinity-Mediated Selection

Target oligonucleotides were dissolved in a high-salt immobilization buffer (1.5 M NaCl, 20 mM 4-(2-hydroxyethyl)-1-piperazineethanesulfonic acid (HEPES,) pH 7.5). This solution was then incubated with streptavidin T1 (Invitrogen) magnetic beads, which had been pre-equilibrated in the same buffer. Unbound target was washed away with three washes of four-column volumes of selection buffer (20 mM HEPES, 300 mM KCl, 10 mM MgCl_2_, 0.02% Tween-20, 1 mg/mL sssDNA, pH 7.5). The immobilized oligonucleotides were allowed to equilibrate in the selection buffer for 40 min to permit G-quartet formation [13]. The combined set of 33 DNA-encoded libraries was dissolved (30 pmol of each in a volume of 60 µL) in the selection buffer. After incubation with the immobilized target oligonucleotides for 1 h, the matrix was washed five times with 200 µL of the selection buffer and then eluted using 10 mM of NaOH. The eluate was immediately neutralized. The output from the first cycle of affinity-mediated selection was used as the input for a second cycle with the same protocol, using freshly immobilized target oligonucleotides. An additional no-target selection was conducted in the absence of any added oligonucleotide target, and each selection was conducted in duplicate with a parallel library, within which the linkage between the encoded compound and the encoding DNA had been cut by restriction digest in order to control for DNA-mediated binding events such as hybridization. Capture of the biotinylated oligonucleotides was monitored by elution at 100 °C for 3 min in deionized water, followed by UV shadowing on denaturing polyacrylamide gel electrophoresis (PAGE.) The output of the second round of affinity-mediated selection was amplified using PCR with primers that introduced Illumina READ1 and READ2 sequences and submitted for sequencing using an Illumina 2500 instrument in high-output mode. In total, 493,099,795 single-end reads were generated for all 33 libraries under all conditions, of which 1,027,749 qualifying reads were derived from Library A and 672,851 qualifying reads were derived from Library B, each specifically after selection against the c-myc oligonucleotide target.

### 2.4. Selection Output Analysis

**Figure 2:**
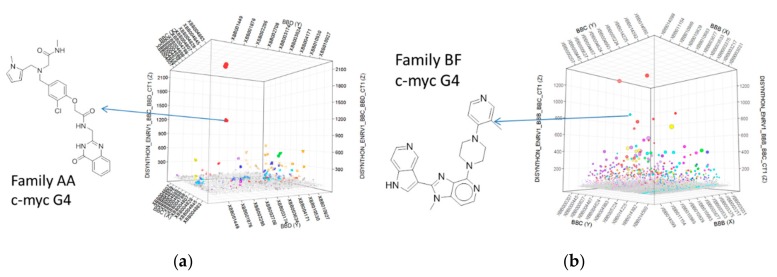
3D scatter plots showing individual enriched features against the c-myc G4 oligonucleotide. These were used to inform the design of compounds for resynthesized without DNA attached. (**a**) Library A, with Family AA in red; (**b**) Library B, with Family BF in cerulean blue. Arrows indicate which of the enriched features represent the resynthesized active compounds. The *x* and *y* axes represent individual building blocks at the indicated cycles of synthesis. The *z* axis represents a statistical metric of enrichment significance (ENRv1) for each disynthon (negative log_10_ of the asymptotic significance value).

**Figure 3:**
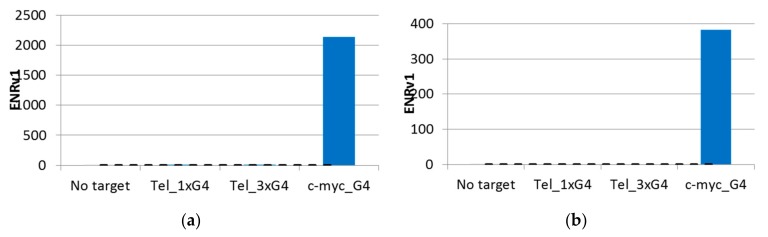
Profiles indicating the relative enrichments of specific compounds across different selections: (**a**) Compound **1**; (**b**) Compound **2**. These compounds were enriched only against the c-myc G4 target and were not enriched against the off-targets or the no-target control. The *y* axis represents a metric of enrichment significance (ENRv1) for each disynthon (negative log_10_ of the asymptotic significance value).

**Figure 4:**
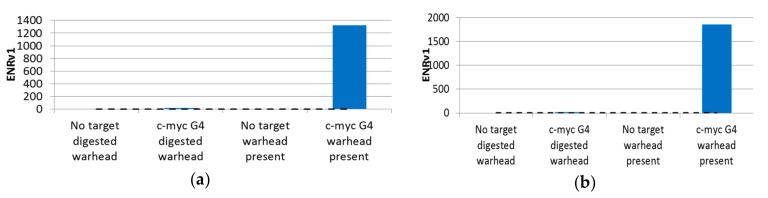
Relative enrichment of compounds with and without a target and with and without an encoded chemical entity: (**a**) Family AA; (**b**) Family BF. Enrichment was observed to be both target- and encoded chemistry-dependent: When the encoded chemical entity was cleaved from the library, no enrichment was observed. Thus, we ruled out encoding DNA–target DNA complementarity as being responsible for the observed enrichment in Figure 1; Figure 2. The *y* axis represents a metric of enrichment significance (ENRv1) for each disynthon (negative log_10_ of the asymptotic significance value).

Individual sequence reads were translated back into the chemical information they encoded, and statistical parameters were calculated for all disynthon pairs within each library. Individual libraries were then visualized using 3D scatter plots with building block identifiers defining the *x* and *y* axes, and an enrichment significance metric (ENRv1) for each disynthon (negative log_10_ of the asymptotic significance value) was used to define the *z* axis. Libraries A and B are shown in Figure 2a,b, respectively, with each point representing a fully elaborated instance (library member) and each enriched jittered cluster representing a contiguous pair of building blocks (disynthon). Each point was sized by normalized count. These plots were then used to identify compounds (Figure 2) for resynthesized without -DNA attached. Profiles were then determined by measuring the extent of enrichment of each enriched building block combination in each of the parallel selections (Figure 3a,b. Similarly Figure 4 indicates that Families AA and BF do not enrich if the linkage between the encoding oligonucleotides and the encoded chemical entity is cleaved by restriction digest.

### 2.5. Compound Design and Synthesis

**Figure 5:**
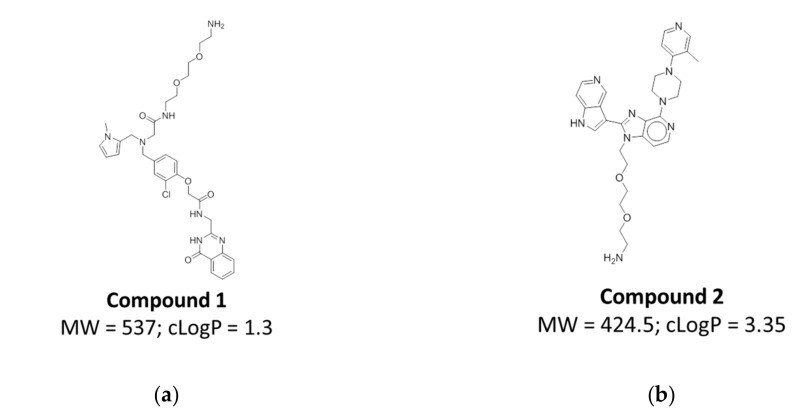
Two compounds synthesized based on their enrichment profiles: (**a**) Compound **1** from Family AA in Library A; (**b**) Compound **2** from Family BF in Library B. Each compound was synthesized with a diethylene glycol-ethyl amino linker at the DNA attachment site, with the amino group available for modifications (e.g., biotinylation) of the compounds.

Based on the individual profiles and the physicochemical values of the indicated chemical matter, we chose individual compounds of interest to be synthesized without DNA attached. These compounds were resynthesized with a diethylene glycol ethylamine handle to allow for chemical functionalization, e.g., of a fluorophore or biotin (Figure 5, Figures S3 and S4). Synthesis protocols for these compounds are shown in Schemes S1 and S2 respectively.

### 2.6. Affinity Assessment Using Surface Plasmon Resonance

In order to measure the affinity of the resynthesized off-DNA compounds with their cognate oligonucleotide targets, we performed surface plasmon resonance (SPR) measurements using a Biacore 3000. The compounds were synthesized with a short linker attached, which was then biotinylated prior to immobilization on the streptavidin-coated chip surface with the target oligonucleotides then passing over this surface in solution. 

**Figure 6:**
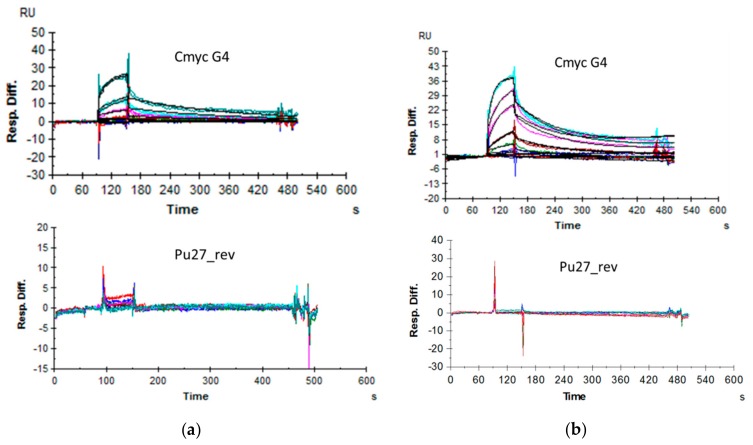
Surface Plasmon Resonance data. Sensorgrams recorded using a Biacore 3000. Biotinylated compounds were immobilized on a streptavidin chip, with solution-phase nonbiotinylated DNA as the analyte. (**a**) Compound **1**; c-myc G4 Langmuir 1:1 k_a_ = 4.25 × 10^3^; k_d_ = 4.46 × 10^−3^, *Rmax* 15.4, *K*_D_ = 1.05 uM, chi^2^ = 0.643; Pu27rev no binding; (**b**) Compound **2**; c-myc G4 Langmuir 1:1 k_a_ = 2.83 × 10^4^; k_d_ = 9.29 × 10^−3^, *Rmax* 28, *K*_D_ = 328 nM, chi^2^ = 0.511; Pu27rev no binding. Measurements were performed in duplicate. Color-coded concentrations (in μM): 0 (dark blue), 0.01 (light red), 0.03 (light blue), 0.1 (dark green), 0.3 (dark red), 1 (light magenta), 3 (light cyan), 10 (dark cyan).

Initial SPR experiments were performed with biotinylated oligonucleotides immobilized on a streptavidin chip surface and with compounds as solution-phase analytes. This approach was hampered by multiple technical challenges, including the necessity of using high salt concentrations to permit immobilization of the oligonucleotides and the relatively low signal strengths that resulted from the low masses of the small-molecule analytes binding to the immobilized targets because of their corresponding small influence upon the evanescent wave. Instead, we ultimately took advantage of the fact that since DNA-encoded chemical library outputs are discovered in a linked form, they are readily resynthesized with a chemically functionalized linker installed that may then be used to immobilize them upon a surface while maintaining their binding abilities. We generated the small molecules with short linkers, biotinylated them, and immobilized them directly on a streptavidin-coated surface, with the nucleic acids as solution-phase analytes. This approach generates higher signal strengths because the larger mass changes that result from binding events have a correspondingly larger effect upon the evanescent wave. This approach also has the advantage of being orthogonal to the affinity-mediated discovery screen and is therefore less likely to suffer from the same artifacts. The compounds were synthesized with a short linker, which was then biotinylated by reaction with biotin-NHS (Pierce Biotechnology, Waltham, MA, USA). These biotinylated compounds were then captured on the streptavidin-coated chip surface. We studied interactions with several different G-quartet structures and other oligonucleotides using this method. We discovered that both Compounds **1** and **2** bind to G-quartet targets with very little signal observed for binding to the unstructured control DNA. Figure 6 shows sensorgrams obtained using this approach. The interaction of c-myc G4 with immobilized Compound **2** could be fit as a Langmuir 1:1 (*K*_D_ 328 nM). We also discovered that the oligo Pu21_1234, which spans the first four G-quartets of the c-myc G4 Pu-27 construct [8], had an even higher affinity (*K*_D_ 59 nM), whereas binding to Pu21_2345 demonstrated a somewhat lower affinity with Compound **2**, with a *K*_D_ of 190 nM. The lower apparent affinity with the full-length 27-mer C-myc G4 could be explained by the presence of multiple conformational states, as was expected for this oligonucleotide. Similarly, Compound **1** demonstrated binding, with a *K*_D_ of 1.06 µM, to c-myc G4, with higher-affinity interactions with both Pu21_1234 (*K*_D_ of 328 nM) and Pu21_2345 (*K*_D_ of 308 nM) not appearing to discriminate between the two loop isomers. While both loop isomers contained the same total loop length, the two major loop conformers formed in C-myc G4 1234 appeared to be markedly less stable than the major 1:2:1 loop conformer G-quadruplexes formed in C-myc G4 2345 of NHE III1 in K^+^ solution, which may suggest a physiological role for C-myc G4 2345 in c-myc expression regulation [8]. Table 2 shows affinities as determined by SPR. The relative selectivity of interaction between the small molecules and other G-quartet target sequence recognition appeared to be low, with a 3–10-fold lower *K*_D_ for the cognate target interaction when compared to interactions with other G-quartet target sequences. For the highest-affinity interaction with c-myc Pu21_1234 of 59 nM, a 13–100-fold selectivity was observed when compared to other G-quartet target sequences. 

### 2.7. PCR-Stop Assay

**Figure 7:**
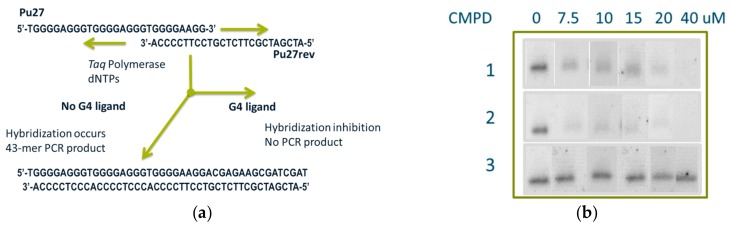
PCR-stop assay. (**a**) Schematic of the assay: Priming of Pu27 c-myc G4 template amplification by Pu27rev oligonucleotides, which was complementary to the 3′ end portion of C-myc G4. Hybridization occurred if no G-quartet structure formed and a 43-mer extended primer was generated. If the G-Quartet was stabilized by the added small-moleculethen hybridization and subsequent primer extension were inhibited. (**b**) A dose-dependent reduction in quantity of primer extension product was observed with Compounds **1** and **2**, whereas no inhibition was observed with Compound **3**, which was used as a negative control (Figure S1). Full gels are presented in Figure S5.

Compounds were also ranked for their affinity to G-quartet-containing oligonucleotides using a PCR-stop assay [29,30]. In this homogeneous assay, oligonucleotides are utilized as templates for primer extension using a PCR primer that is complementary to a terminal G-repeat. Polymerase-mediated extension results in the formation of a 43-base pair double-stranded PCR product that may be further amplified under PCR conditions and ultimately detected. In the presence of a ligand that stabilizes test oligonucleotides into a G-quartet structure, the annealing of the primer and the downstream extension is inhibited, resulting in a reduction in the amount of PCR product. Oligomer Pu27 c-myc G4 was incubated in the presence of the complementary strand Pu27rev primer and thermally cycled 30 times with increasing concentrations of the different small molecules (Figure 7a). Using this assay, we observed that Compounds **1** and **2** inhibited the formation of primer extension products consistent with their ability to interact with these oligonucleotides in solution. As a negative control, we used Compound **3** (Figure S1), which did not bind the C-myc G4 oligonucleotide and was observed to have no effect on its PCR amplification at concentrations of up to up to 40 uM (Figure 7b).

### 2.8. Cell Culture Experiments

**Figure 8:**
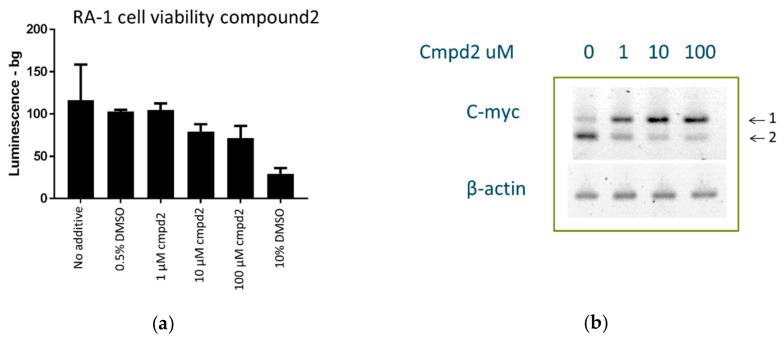
Viability of RA 1 cells (Burkitt’s lymphoma, dependent on c-myc): (**a**) 48-h cell viability in the presence of different concentrations of Compound **2** as determined by Cell Titer-Glo assay (Promega); (**b**) RT-PCR of c-myc expression in RA 1 cells after 24 h of treatment with different concentrations of Compound **2**. Here, 1: Full-length PCR product; 2: Primer-dimer. Full gels are presented in Figure S6.

The c-myc gene is translocated to one of the immunoglobulin loci in all Burkitt’s lymphomas. The typical Burkitt’s lymphoma-derived RAMOS (RA 1) cell line (ATCC^®^ CRL-1596^™^, ATCC, Manassas, VA, USA) contains such a translocation [19]. It has been previously demonstrated that the G-quartet binder TMPyP4 lowers MYC expression in RA 1 cells, which maintain G4-mediated transcription control of MYC in the translocated allele [18,19,31,32]. We used RA 1 cells as a model system to study the effect of Compound **2** on cell proliferation and c-myc expression. Cells treated with Compound **2** did not display significant toxicity at 1 uM after 48 h of a luminescence assay: A very modest 10–15% inhibition of cell growth was observed at 10–100 uM, and 0.5% Dimethyl Sulfoxide (DMSO) had no effect, whereas 10% DMSO caused a 65–70% inhibition of cell growth (Figure 8a). Using reverse transcription from total RNA followed by PCR amplification with gene-specific primers, we observed that increasing concentrations of Compound **2** produced a stimulatory effect upon c-myc expression, which therefore suggested a direct interaction between the compound and the c-myc promoter. This experiment was repeated twice on different days and with different cells and batches of the compound to rule out artifacts (Figure 8b). In addition, β-actin assay was used as a control, and it did not respond to treatment of the cells with Compound **2**.

## 3. Discussion

We used DNA-encoded chemical libraries and affinity-mediated selection to discover small-molecule binders to G-quartet-forming DNA oligonucleotide targets, including for a c-myc-derived sequence. These compounds exhibited nanomolar to low-micromolar K_D_ values and were highly selective over non-G-quartet-forming DNA oligonucleotides, while showing some selectivity versus other G-quartet-forming DNA oligonucleotides. This was all the more remarkable in view of the fact that the library deck utilized was not designed with nucleic acid targets in mind and is merely the product of the combinatorial conjugation of low-mass commercially available building blocks sourced without regard to any particular target or scaffold. It was relatively straightforward to find small molecules that specifically bind to G-quartet-forming oligonucleotides and not to other DNA structures. The discovery of molecules that are specific to particular G-quartets remains somewhat challenging. One simple explanation may be that all G-quartet structures are highly similar and there are only limited ways a small molecule ligand can bind to them [13]. Affinities were determined using SPR and show values as low as the double-digit nM range. Target engagement in solution was indicated by showing the inhibition of primer extension of a G-quartet-containing oligonucleotide template, and target engagement in a cellular environment was consistent with our observation of a dose-dependent increase in c-myc expression under control of a G-quartet-containing promoter after 24 h. We hope that this study will inspire others to consider the application of DNA-encoded chemical libraries to targets derived from other nucleic acid structural classes and to apply this methodology to the discovery of new therapeutic agents.

## 4. Materials and Methods

### 4.1. Library Synthesis

Libraries A and B were synthesized by a split and pool procedure in which both a chemical step and a DNA tagging step occurred in each split. The general procedures for DNA-encoded library synthesis have been described before [3,4,5,6,7,8,27,28]. Library A was a 3-cycle library, and its synthesis was initiated with the installation of bromoacetic acid onto the linker. Then the material was split into 1024 wells. The first cycle (B) was performed by the installation of 1024 primary amines or anilines using SN2. Each split of cycle B was tagged with 1 of 1024 unique oligonucleotides and pooled. Then the library was split into 85 wells, into each of which 1 of 85 formyl acids was installed by reductive alkylation (cycle C). Each split of cycle B was tagged with 1 of 85 unique oligonucleotides and pooled. Finally, the library was split again to install 2500 amines by acylation (cycle D). Each split of cycle D was tagged with one of 2500 unique oligonucleotides and pooled. Library A totaled 217.6 million linear molecules (Figure 1a). Library B had 1.3 million individual compounds and was comprised of two diversity steps displayed on an imidazopyrimidine core (Figure 1b). Synthesis was initiated with a nitro-chloro-pyridine core, to which 1051 secondary amines were installed by S_N_Ar at cycle B. The material was pooled, and the nitro group was reduced. At the following cycle, 1213 aldehydes were installed by using oxidative cyclization. The library was tagged in a similar fashion to Library A. Library B totaled 1,274,863 individual compounds. Each step of library synthesis was monitored by LCMS, and each library was HPLC-purified on a Gilson HPLC (Middletown, WI, USA) after each split and pool cycle.

### 4.2. Oligonucleotide Synthesis

All oligonucleotide targets were synthesized by Integrated DNA Technologies (Coralville, IA, USA) and were HPLC-purified. All targets were LCMS-validated in-house (Figure S2).

### 4.3. Affinity-Mediated Selection

Synthetic biotinylated DNA oligos were reconstituted in water to a 1-mM concentration. Magnetic beads (Streptavidin T1, Invitrogen) were equilibrated in an immobilization buffer (1.5 M NaCl, 20 mM HEPES, pH 7.5). For each target, 50 µL of the beads were aliquoted, and 20 pmol of biotinylated oligonucleotide was added in immobilization buffer. The oligonucleotides were allowed to capture for 30 min at room temperature and were then washed using the same buffer. Selection buffer was then added (HEPES (20 mM), KCl (300 mM), MgCl_2_ (10 mM), Tween-20 (0.02%), sssDNA (1 mg/mL) at pH 7.5), washed 3 times, and equilibrated in the same buffer for 40 min. Maintenance of the c-myc G-quartet integrity required a sufficient amount of K^+^ in the solution, so we used a selection buffer containing 300 mM of KCl. The beads were then divided into two 25-µL aliquots. To the first aliquot, 1 nmol of the combined library deck was applied to the beads and incubated for 1 h at room temperature. Then it was subjected to five 200-µL washes and eluted by 25 µL of 10-mM NaOH, followed by immediate neutralization with 15 µL of 1-M Tris HCl (pH 7.0). The sample was adjusted to the selection buffer and was reapplied to the second aliquot of the same beads with the same immobilized target. The procedure was repeated. The output of the second round of affinity-mediated selection was amplified using PCR with primers that introduced Illumina READ1 and READ2 sequences and submitted for sequencing using an Illumina 2500 instrument in high-output mode: 493,099,795 single-end reads were generated in total for all 33 libraries under all conditions, of which 1,027,749 qualifying reads were derived from Library A and 672,851 qualifying reads were derived from Library B, each specifically after selection against the c-myc oligonucleotide target.

### 4.4. Surface Plasmon Resonance Experiments

SPR experiments were performed on a Biacore 3000. Streptavidin (SA) sensor chips and instrument-specific disposables were purchased from GE Healthcare (Marlborough, MA, USA). 

#### 4.4.1. Surface Plasmon Resonance

##### Compound Biotinylation

Biotinylated compounds were prepared through the reaction of 10 nmol of each of Compounds **1** and **2** with 1 equivalent biotin-NHS ester (Pierce) in 20 µL DMSO in the presence of 1% isopropyl ethyl amine. The reaction was monitored by small-molecule LCMS, and quantitative reaction yields were observed. To quench the leftover reactive NHS reagent, ethanolamine was added to a final concentration of 1%. The product was used without further purification. It was diluted into SPR immobilization buffer (20 mM HEPES, 200 mM KCl, 5 mM MgCl_2_; 1% DMSO, 0.005% P-20, pH 7.5) to achieve a 1-uM concentration and then injected at a flow rate of 5 µL/min over the flow cell until 140–190 RU was captured.

##### Affinity Assessment through SPR

Oligonucleotide samples were dissolved in water to concentrations of 10 mM. Dilutions were made into SPR running buffer (20 mM HEPES, 200 mM KCl, 5 mM MgCl_2_; 1% DMSO, 0.005% P-20, pH 7.5). The oligonucleotide concentrations tested ranged from 0.03 to 10 uM. Analyte solutions were injected for 60 s at 30 µL/min, and dissociation was monitored for 300 s. Blanks were run between each cycle, and an injection wash step was incorporated to minimize sample carryover. Data were processed using reference subtraction (the response from the reference cell was subtracted from the sample flow cell). Binding affinities were determined using Bioevaluation software by plotting the sensorgrams obtained for different ligand concentrations and fitting these to 1:1 Langmuir kinetics.

### 4.5. PCR-Stop Assay

The stabilization of G-quartet structures by specific ligands was investigated by a PCR-stop assay [29,30] using a test oligonucleotide and a complementary oligonucleotide that partially hybridizes to the last G-repeat of the test oligonucleotide. Sequences of the test oligonucleotides (Pu27 c-myc G4) and the corresponding complementary sequence (Pu27rev) used here are presented in Figure 4. Assay reactions were performed in a final volume of 25 µL in Platinum Supermix (ThermoFisher) supplemented with 50 mM of KCl: 0.5 uM of each of the two oligonucleotides and the indicated amount of the ligand. Reaction mixtures were incubated in a thermocycler with the following cycling conditions: 94 °C for 2 min, followed by 30 cycles of 94 °C for 30 s, 58 °C for 30 s, and 72 °C for 30 s. Amplified products were resolved on 4% agarose gels (Invitrogen) stained with ethidium bromide. The gels were analyzed on an Alpha Innotech system using transillumination.

### 4.6. Cell Viability Assay

Cell proliferation was assessed using the CellTiter-Glo^®^ Luminescent Cell Viability Assay (Promega, Madison, WI, USA). Cells were treated in a 96-well plate in triplicate with 1–100 μM of Compound **2** dissolved in medium containing 0.5% DMSO for 48 h (10^5^ cells/well). Additional individual no-compound controls were conducted with 0%, 0.5%, and 10% DMSO. Equal volumes of freshly prepared CellTiter-Glo^®^ reagent (100 μL) were added to each well, and the plate was incubated at room temperature on an orbital shaker to lyse the cells. An additional 10 min incubation at room temperature was performed to stabilize the signal, and the plate was read on a Tecan plate reader in luminescence mode.

### 4.7. RT-PCR

Two million RA 1 cells were seeded in a 6-well plate and treated with 1, 10, or 100 uM of Compound **2** dissolved in RPMI + 0.5% DMSO for 24 h. As a control, 0.5% DMSO in RPMI was used. After incubation, the cells were collected and total RNA was extracted from them using TriZol reagent according to the manufacturer’s instructions. The extracted total RNA was then purified on an RNeasy column (Qiagen) with a DNAse I digest. This two-step process achieved completely DNA-free samples. RNA purity was determined through the A260/A280 ratio and quantified through A260. To prepare cDNA, 20 ng of total RNA was incubated in a reaction mix containing 1× First Strand Synthesis Buffer, 5.5 mM MgCl_2_, a mixture of all four deoxy nucleotide triphosphates (dNTP’s) (500 uM of each dNTP), 2.5 uM of random hexamer and oligo (dT) primers, 0.2 units/µL RNAse inhibitor, and 1.25 units/µL Superscript IV (Invitrogen) Reverse Transcriptase. The reaction mixture was first incubated for 10 min at 25 °C to maximize primer-RNA template binding. Reverse transcription of RNA to generate cDNA was performed with a 20-min incubation at 55 °C immediately followed by a 5-min incubation at 95 °C to stop the reaction and denature the cDNA. PCR was performed for a uniform amount of cDNA (platinum, Invitrogen) with β-actin and c-myc specific primers, as indicated below [22]: c-myc forward primer 5′-CGTCTCCACACATCAGCACAA-3′ and reverse primer 5′-TCTTGGCAGCAGGATAGTCCTT-3′, β-actin forward primer 5′-TGCCGACAGGATGCAGAAG-3′ and reverse primer 5′-CTCAGGAGGAGCAATGATCTTGA-3′. The c-myc primers annealed to the third exon of the c-myc gene, producing a 67-bp amplicon, while the b-actin primers produced a 75-bp amplicon. Each PCR reaction was pre-incubated at 95 °C for 3 min, followed by 36 thermal cycles at 95 °C for 30 s, 50 °C for 30 s, and 72 °C for 30 s. PCR products were resolved on a 4% agarose gel (Invitrogen) stained by ethidium bromide.

## Figures and Tables

**Table 1:** DNA sequences used in this study.

Name	Sequence	Description
Tel1xG4	5′-AAAGGGTTAGGGTTAGGGTTAGGGAA-3′	Single G-quartet forming four telomere repeats
Tel1xG4-biotin	5′-Biotin-C18-C18-AAAGGGTTAGGGTTAGGGTTAGGGAA-3′	Biotinylated single G-quartet forming four telomere repeats
Tel3xG4	5′-AAAGGGTTAGGGTTAGGGTTAGGGTTAGGGTTAGGGTTAGGGTTAGGGTTAGGGTTAGGGTTAGGGTTAGGGTTAA-3′	Triple G-quartet forming 12 telomere repeats
Tel3xG4-biotin	5′-Biotin-C18-C18-AAAGGGTTAGGGTTAGGGTTAGGGTTAGGGTTAGGGTTAGGGTTAGGGTTAGGGTTAGGGTTAGGGTTAGGGTTAA-3′	Biotinylated triple G-quartet forming 12 telomere repeats
C-mycG4 (Pu-27)	5′-TGGGGAGGGTGGGGAGGGTGGGGAAGG-3′	C-myc G4
Biotin-C-mycG4 (biotin-Pu-27)	5′-Biotin-C18-C18-TGGGGAGGGTGGGGAGGGTGGGGAAGG-3′	Biotinylated C-myc G4
Pu27rev	ATCGATCGCTTCTTCGTCCTTCCCCA	Non-G-quartet control sequence, Complement to Pu-27 for PCR-stop assay
c-myc_G4_1234	5′-TGGGGAGGGTGGGGAGGGTGG-3′	3′-end truncated C-myc G4
c-myc_G4_2345	5′-GAGGGTGGGGAGGGTGGGGAA-3′	5′-end truncated C-myc G4
C-kit1	5′-AGGGAGGGCGCTGGGAGGAGGG-3′	First G-quartet of C-kit promoter
C-kit2	5′-GGGCGGGCGCGAGGGAGGGG-3′	Second G-quartet of C-kit promoter

**Table 2:** *K*_D_ values for reported compounds as determined by Surface Plasmon Resonance.

Target	Compound 1 ^1^	Compound 2 ^1^
C-myc G4	1.05 µM	328 nM
C-myc G4 1234	338 nM	59 nM
C-myc G4 2345	308 nM	197 nM
C-kit1 G4	1.1 uM	807 nM
C-kit2 G4	1.7 uM	1.4 uM
Telomere G4	8.4 uM	5.25 uM
HP006	NB	NB
Pu27rev	NB	NB

^1^ Biotinylated and immobilized. NB is No Binding.

## Supplementary Materials

The following are available online at https://www.mdpi.com/1420-3049/24/10/2026/s1, Figure S1: Compound **3** used as a negative control in the PCR-stop assay. Figure S2: LCMS of the DNA oligonucleotides used as targets for the affinity-mediated selection. Figure S3: Scheme of the synthesis of Compound **1**. Figure S4: Scheme of the synthesis of Compound **2**. Figure S5: Full gels of the PCR-stop assay. Figure S6: Full gels of the RT-PCR reactions derived from cells treated with different concentrations of Compound **2**. Scheme S1: Synthesis of Compound **1**. Scheme S2: Synthesis of Compound **2**.
