# Supplementary material for: Novel Nucleic Acid Binding Small Molecules Discovered Using DNA-Encoded Chemistry
Source: Molecules. 2019 May 27;24(10):2026. doi: 10.3390/molecules24102026 (PMC6572338; doi:10.3390/molecules24102026)
Supplement: Supplementary file 1 [file molecules-24-02026-s001.pdf]

molecules

MDPI

Supplementary Information

Novel Nucleic Acid Binding Small Molecules Discovered using DNA-Encoded Chemistry

Alexander Litovchick [1], Xia Tian [2], Michael I. Monteiro [1], Kaitlyn Kennedy [1], Marie-Aude Guié [1], Paolo A. Centrella [1], Ying Zhang [1], Matthew A. Clark [1] and Anthony D. Keefe [1,*]

[1] X-Chem Pharmaceuticals, Waltham, MA, USA; alitovchick@x-chemrx.com (A.L.); mmonteiro@x-chemrx.com (M.I.M.); Kaitlyn.kennedy1288@gmail.com (K.K.); mguie@x-chemrx.com (M.-A.G.); pcentrella@x-chemrx.com (P.A.C.); yzhang@x-chemrx.com (Y.Z.); mclark@x-chemrx.com (M.A.C.)

[2] Arrakis Therapeutics, Waltham, MA, USA; xia2tian@hotmail.com

* Correspondence: keefe@x-chemrx.com

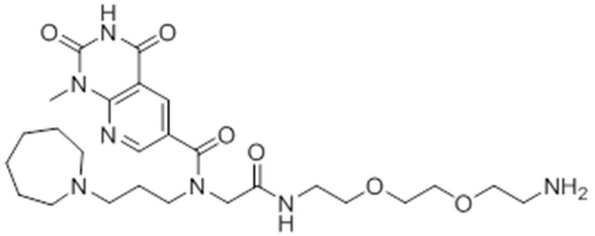

Figure 1. Compound 3 used as a negative control in the PCR-stop assay.

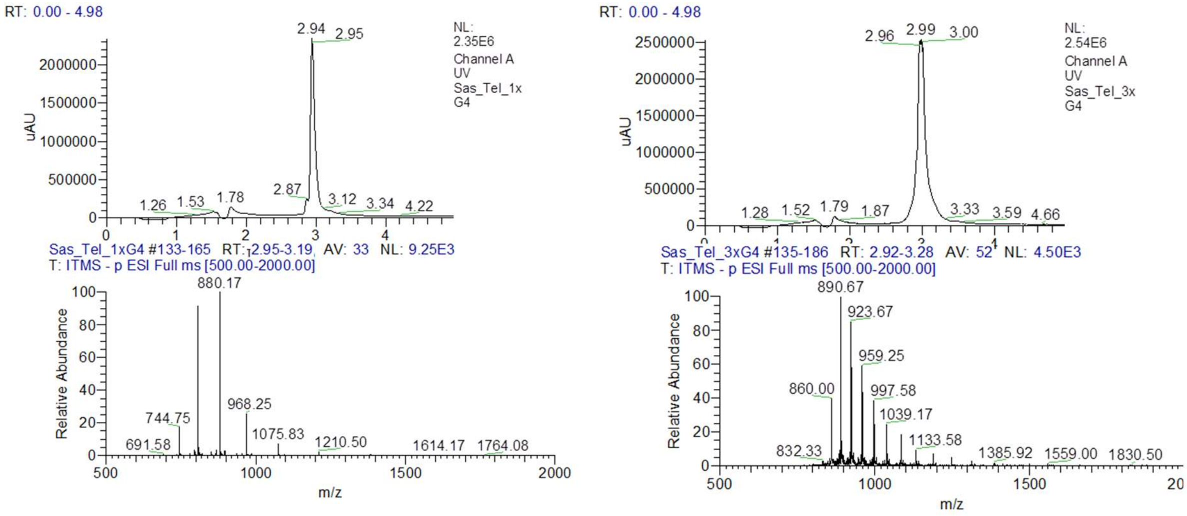

(**a**) (**b**)

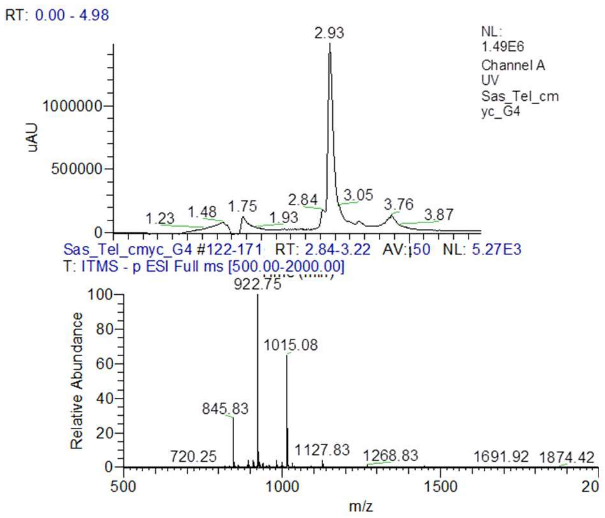

(**c**)

Figure S2. LCMS of the DNA oligonucleotides used as targets for affinity-mediated selection (**a**) Tel1xG4-biotin MW 9,683.0 (calc. 9,693.7); (**b**) Tel-3xG4-biotin MW 24,966.2 (calc. 24,967.5); (**c**) Biotin-c-myc G4 MW 10,161.2 (calc. 10,161.9)

Figure S3. Scheme of the synthesis of Compound 1.

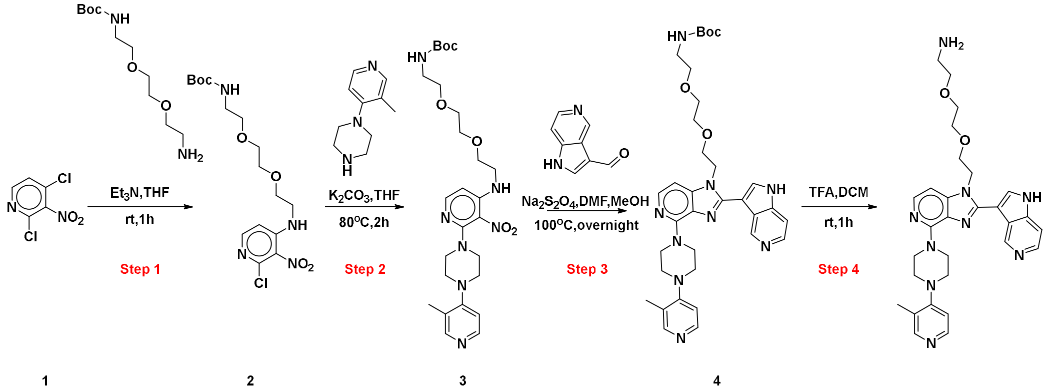

Figure S4. Scheme of the synthesis of compound 2.

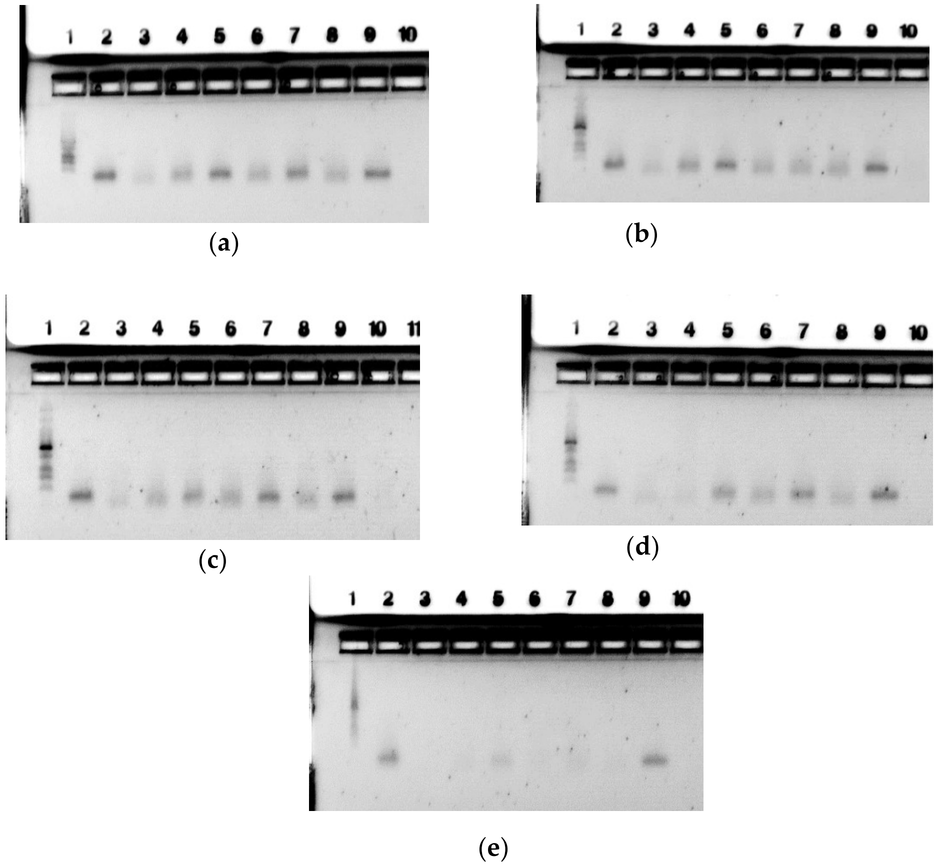

Figure S5. Full gels showing the dose-dependent reduction in quantity of PCR product derived from template containing G-quartet sequence in the presence of compounds 1 and 2 whereas no inhibition was observed by compound 3, used as a negative control (Figure S1). Each gel: 1- marker, 2- control reaction without any compounds. Compound 1 – lane 8, Compound 2- lane 3, Compound 3- lane 9. Lanes 4, 5, 6, 7- different compounds, which we do not describe in this paper. Each compound is at (**a**) 7.5 µM, (**b**) 10 µM, (**c**) 15 µM, (**d**) 20 µM, (**e**) 40 µM.

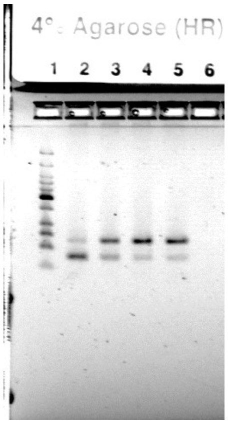

(**a**)

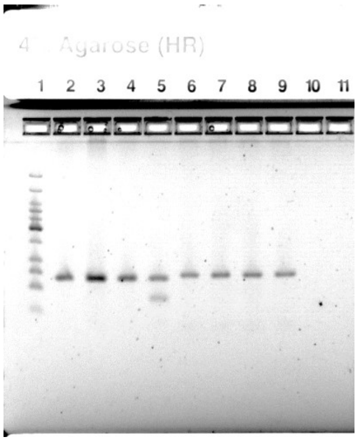

(**b**)

Figure S6. Full gels of the RT-PCR reactions from the cells treated with different concentrations of Compound 2: (**a**) c-myc, Lanes: 1- low mw DNA ladder (NEB), 2- No compound 2, 3- 1 μM, 4- 10 μM, 5-100 μM of compound 2 (**b**) b-actin: Lanes: 1- low mw DNA ladder (NEB), 6- No compound 2, 7- 1 μM, 8- 10 uM, 9-100 μM of compound 2; Lanes 2-5- RT-PCR analysis of a different gene, not described in the paper.

Scheme S1: Synthesis of Compound 1.

Step1: Into a 25-mL round-bottom flask (1 atm), was placed 3-chloro-4-hydroxybenzaldehyde (1.1 g, 7.03 mmol, 1.00 equiv), N,N-dimethylformamide (10 mL), potassium carbonate (1.5 g, 10.85 mmol, 1.60 equiv), methyl 2-bromoacetate (1.4 g, 9.15 mmol, 1.30 equiv). The resulting solution was stirred for 2 h at room temperature. The reaction was then quenched by the addition of 20 mL of water. The resulting solution was extracted with 2x20 mL of ethyl acetate and the organic layers combined. The resulting mixture was washed with 2x20 mL of brine. The mixture was dried over anhydrous sodium sulfate and concentrated under vacuum. This resulted in 1.5 g (93%) of methyl 2-(2-chloro-4-formylphenoxy)acetate as a white solid.

m/z (ES^+) $[M+H]^+$ = 229; HPLC tR= 1.04 min.

Step2: Into a 25-mL round-bottom flask (1 atm), was placed methyl 2-(2-chloro-4-formylphenoxy)acetate (1.5 g, 6.56 mmol, 1.00 equiv), methanol (5 mL), water(5 mL), sodium hydroxide (1 g, 25.00 mmol, 4.00 equiv, aqueous solution). The resulting solution was stirred for 1 h at room temperature. The reaction progress was monitored by LCMS. The resulting mixture was concentrated under vacuum. The residue was dissolved in 40 mL of water. The pH value of the solution was adjusted to 3 with hydrogen chloride a.q. (1 mol/L). The resulting solution was extracted with 2x50 mL of ethyl acetate and the organic layers combined. The resulting mixture was washed with 2x50 mL of brine. The mixture was dried over anhydrous sodium sulfate. This resulted in 1.2 g (85%) of 2-(2-chloro-4-formylphenoxy)acetic acid as a white solid.

m/z (ES^+) $[M+H]^+$ = 215; HPLC tR= 0.72 min.

Step 3: Into a 50-mL round-bottom flask (1 atm), was placed 2-(2-chloro-4-formylphenoxy)acetic acid (900 mg, 4.19 mmol, 1.00 equiv), 2-(aminomethyl)-3,4-dihydroquinazolin-4-one dihydrochloride

(1.2 g, 4.84 mmol, 1.20 equiv), HOBT (810 mg, 5.99 mmol, 1.50 equiv), EDCI (1.2 g, 6.26 mmol, 1.50 equiv), sodium bicarbonate (1.2 g, 14.28 mmol, 3.50 equiv), N,N-dimethylformamide (20 mL). The resulting solution was stirred for 1 h at room temperature. The reaction progress was monitored by LCMS. The resulted solution was purified through C18 column (45% ACN, 35 min). This resulted in 1.5 g (96%) of 2-(2-chloro-4-formylphenoxy)-N-[(4-oxo-3,4-dihydroquinazolin-2-yl)methyl]acetamide as a yellow solid.

m/z (ES$^+$) [M+H]$^+$ = 372; HPLC tR= 1.09 min.

Step 4: Into a 100-mL round-bottom flask (1 atm), was placed 2-(2-chloro-4-formylphenoxy)-N-[(4-oxo-3,4-dihydroquinazolin-2-yl)methyl]acetamide (400 mg, 1.08 mmol, 1.00 equiv), AcOH (0.5 mL), $NaBH_3CN$ (105 mg, 1.67 mmol, 1.50 equiv), methanol (50 mL), (1-methyl-1H-pyrrol-2-yl)methanamine (150 mg, 1.36 mmol, 1.30 equiv). The resulting solution was stirred for 6 h at room temperature. The resulting mixture was concentrated under vacuum. The residue was purified through C18 column (MeOH, 3 min). This resulted in 400 mg (80%) of 2-[2-chloro-4-([[(1-methyl-1H-pyrrol-2-yl)methyl]amino]methyl)phenoxy] -N-[(4-oxo-3,4-dihydroquinazolin-2-yl)methyl]acetamide as a white solid.

m/z (ES$^+$) [M+H]$^+$ = 466; HPLC tR= 0.95 min.

Step 5: Into a 50-mL round-bottom flask (1 atm), was placed 2-[2-chloro-4-([[(1-methyl-1H-pyrrol-2-yl)methyl]amino]methyl)phenoxy]-N-[(4-oxo-3,4-dihydroquinazolin-2-yl)methyl]acetamide (100 mg, 0.21 mmol, 1.00 equiv), potassium carbonate (41 mg, 0.30 mmol, 1.50 equiv), N,N-dimethylformamide (20 mL), methyl 2-bromoacetate (30 mg, 0.20 mmol, 1.00 equiv). The resulting solution was stirred for 24 h at room temperature. The reaction progress was monitored by LCMS. The resulted solution was purified through C18 column (66% ACN, 50 min). This resulted in 50 mg (43%) of methyl 2-([[3-chloro-4-([[(4-oxo-3,4-dihydroquinazolin-2-yl)methyl]carbamoyl]methoxy)phenyl]methyl][(1-methyl-1H-pyrrol-2-yl)methyl]amino)acetate as a white solid.

m/z (ES$^+$) [M+H]$^+$ = 538; HPLC tR= 1.02 min.

Step 6: Into a 25-mL round-bottom flask (1 atm), was placed methyl 2-([[3-chloro-4-([[(4-oxo-3,4-dihydroquinazolin-2-yl)methyl]carbamoyl]methoxy)phenyl]methyl] [(1-methyl-1H-pyrrol-2-yl)methyl]amino)acetate (50 mg, 0.09 mmol, 1.00 equiv), sodium hydroxide (16 mg, 0.40 mmol, 4.00 equiv), water(2 mL), methanol (4 mL). The resulting solution was stirred for 1 h at room temperature. The reaction progress was monitored by LCMS. The resulted solution was acidized with HCl (aq.). Solvent was removed under vacuo. This resulted in 30 mg (62%) of 2-([[3-chloro-4-([[(4-oxo-3,4-dihydroquinazolin-2-yl)methyl]carbamoyl]methoxy) phenyl]methyl][(1-methyl-1H-pyrrol-2-yl)methyl]amino)acetic acid as a white solid.

m/z (ES$^+$) [M+H]$^+$ = 397; HPLC tR= 1.23 min.

Step 7: Into a 25-mL round-bottom flask (1 atm), was placed 2-([[3-chloro-4-([[(4-oxo-3,4-dihydroquinazolin-2-yl)methyl]carbamoyl]methoxy)phenyl]methyl][(1-methyl-1H-pyrrol-2-yl)methyl]amino)acetic acid (30 mg, 0.06 mmol, 1.00 equiv), HOBT (14 mg, 0.10 mmol, 1.70 equiv), EDCI (19 mg, 0.10 mmol, 1.70 equiv), DIEA (20 mg, 0.15 mmol, 2.50 equiv), N,N-dimethylformamide (5 mL), tert-butyl N-2-[2-(2-aminoethoxy)ethoxy]ethylcarbamate (25 mg, 0.10 mmol, 1.70 equiv). The resulting solution was stirred for 2 h at room temperature. The reaction progress was monitored by LCMS. The resulted solution was purified through C18 column (100% ACN + TFA). This resulted in 40 mg (93%) of tert-butyl N-[2-(2-[2-[2-([[3-chloro-4-([[(4-oxo-3,4-dihydroquinazolin-2-

yl)methyl]carbamoyl]methoxy) phenyl]methyl][(1-methyl-1H-pyrrol-2-yl)methyl]amino)acetamido]ethoxy]ethoxy)ethyl]carbamate as a yellow solid.

m/z (ES^+) $[M+H]^+$ = 754; HPLC tR= 0.89 min.

Step 8: Into a 10-mL round-bottom flask (1 atm), was placed tert-butyl N-[2-(2-[2-[2-([[3-chloro-4-([[(4-oxo-3,4-dihydroquinazolin-2-yl)methyl]carbamoyl]methoxy)phenyl]methyl][(1-methyl-1H-pyrrol-2-yl)methyl]amino)acetamido]ethoxy]ethoxy)ethyl]carbamate (50 mg, 0.07 mmol, 1.00 equiv), a solution of hydrogen chloride in dioxane (4M, 5 mL). The resulting solution was stirred for 2 h at room temperature. The reaction progress was monitored by LCMS. The resulting mixture was concentrated under vacuum. The crude product was purified by Prep-HPLC with the following conditions (2#-AnalyseHPLC-SHIMADZU(HPLC-10)): Column, XSelect CSH Prep C18 OBD Column,, 5um,19*150mm; mobile phase, Water(0.1% FA) and ACN (5.0% ACN up to 24.0% in 7 min); Detector, uv 254nm. This resulted in 11 mg (24%) of N-[2-[2-(2-aminoethoxy)ethoxy]ethyl]-2-([[3-chloro-4-([[(4-oxo-3,4-dihydroquinazolin-2-yl)methyl]carbamoyl]methoxy)phenyl]methyl][(1-methyl-1H-pyrrol-2-yl)methyl]amino)acetamide hydrochloride as a white solid.

m/z (ES^+) $[M+H]^+$ = 654; HPLC tR= 1.43 min.

^{1}H NMR (400 MHz, CD_3OD, 20°C): δ 3.11 (2H, t), 3.45 (2H, t), 3.57-3.72 (12H, m), 4.10 (4H, d), 4.62 (2H, s), 4.78-4.88 (3H, m), 6.06 (1H, t), 6.24 (1H, dd), 6.72 (1H, t), 7.21 (1H, d), 7.40 (1H, dd), 7.52-7.64 (2H, m), 7.70 (1H, d), 7.84 (1H, t), 8.21 (1H, dd), 8.51 (2H, br).

Scheme S2: Synthesis of compound 2.

Step 1: Into a 100-mL round-bottom flask, was placed 2,4-dichloro-3-nitropyridine (1.0 g, 5.21 mmol, 1.00 equiv), tert-butyl 2-(2-(2-aminoethoxy)ethoxy)ethylcarbamate (1.3 g, 5.21 mmol, 1.00 equiv), Et_3N (1.6 g, 15.63 mmol, 3.00equiv), THF (30 mL). The resulting solution was stirred at r.t. for 1 h. The mixture was dissolved in water and extracted with ethyl acetate. The solvent was removed under vacuum. The residue was purified using flash chromatography with the following conditions (Mobile Phase A: Water, Mobile Phase B: ACN; Flow rate: 40 mL/min; Gradient: 0% B to 100% B in 30 min; 254; 220 nm). This resulted in 1.3 g (62%) of tert-butyl 2-(2-(2-(2-chloro-3-nitropyridin-4-ylamino) ethoxy)ethoxy)ethylcarbamate as a white solid.

m/z (ES^+) $[M+H]^-$ = 405; HPLC tR= 1.115 min.

Step 2: Into a 40-mL vial, was placed tert-butyl 2-(2-(2-(2-chloro-3-nitropyridin-4-ylamino)ethoxy)ethoxy)ethylcarbamate (600 mg, 1.50 mmol, 1.00 equiv), 1-(3-methylpyridin-4-yl)piperazine (263 mg, 1.50 mmol, 1.20 equiv), K_2CO_3 (615 mg, 4.50 mmol, 3.00equiv), THF (20 mL). The resulting solution was stirred for 2 h at 80°C. The mixture was diluted in water and extracted with ethyl acetate. The solvent was removed under vacuum. This resulted in 870 mg (crude) of tert-butyl 2-(2-(2-(2-(4-(3-methylpyridin-4-yl)piperazin-1-yl) -3-nitropyridin-4-ylamino)ethoxy)ethoxy)ethylcarbamate as yellow solid.

m/z (ES^+) $[M+H]^-$ = 546; HPLC tR= 0.684 min.

Step 3: Into a 40-mL vial, was placed tert-butyl 2-(2-(2-(2-(4-(3-methylpyridin-4-yl)piperazin-1-yl)-3-nitropyridin-4-ylamino)ethoxy)ethoxy)ethylcarbamate (200 mg, 0.37 mmol, 1.00 equiv), 7-

methoxybenzo[d][1,3]dioxole-5-carbaldehyde (64 mg, 0.44 mmol, 1.20 equiv), $Na_2S_2O_4$ (320 mg, 1.85 mmol, 5.00 equiv), DMF (10 mL) , MeOH (3 mL). The resulting solution was stirred for overnight at 100°C. The mixture was dissolved in water and extracted with ethyl acetate. The solvent was removed under vacuum. This resulted in 250 mg (crude) of tert-butyl 2-(2-(2-(4-(4-(3-methylpyridin-4-yl)piperazin-1-yl) -2-(1H-pyrrolo[3,2-c]pyridin-3-yl) -1H-imidazo[4,5-c]pyridin-1-yl)ethoxy)ethoxy)ethylcarbamate as a yellow oil

(ES, *m*/*z*): m/z (ES^+) $[M+H]^+$ = 642; HPLC tR= 0.833 min

Step 4: Into a 50-mL round-bottom flask, was placed tert-butyl 2-(2-(2-(4-(4-(3-methylpyridin-4-yl)piperazin-1-yl)-2-(1H-pyrrolo[3,2-c]pyridin-3-yl)-1H-imidazo[4,5-c]pyridin-1-yl)ethoxy)ethoxy)ethylcarbamate (250 mg, 0.39 mmol, 1.00 equiv), DCM (8 mL) , TFA (2 mL). The resulting solution was stirred for 1 h at r.t. The solvent was removed under vacuum. The crude product was purified by Prep-HPLC with the following conditions Column: XSelect CSH Prep C18 OBD Column, 5um, 19*150mm; Mobile Phase A: Water (0.05%TFA), Mobile Phase B: ACN; Flow rate: 20 mL/min; Gradient: 7% B to 20% B in 11 min; 254/220 nm; Rt: 8.12 min. This resulted in 39.5 mg (19%) of 2-(2-(2-(4-(4-(3-methylpyridin-4-yl)piperazin-1-yl) -2- (1H-pyrrolo[3,2-c]pyridin-3-yl) -1H-imidazo[4,5-c]pyridin-1-yl)ethoxy)ethoxy)ethanamine as a yellow oil

(ES, *m*/*z*): m/z (ES^+) $[M+H]^+$ = 541; HPLC tR= 1.089 min.

^1H NMR (400 MHz, Methanol-d_4): δ 9.79 (d, *J* = 0.8 Hz, 1H), 8.82 (s, 1H), 8.57 (dd, *J* = 6.7, 0.9 Hz, 1H), 8.35 – 8.25 (m, 2H), 8.16 (dd, *J* = 6.7, 0.8 Hz, 1H), 7.86 (d, *J* = 7.0 Hz, 1H), 7.50 (d, *J* = 7.0 Hz, 1H), 7.33 (d, *J* = 7.0 Hz, 1H), 4.83 (t, *J* = 4.9 Hz, 2H), 4.70 – 4.63 (m, 4H), 4.13 – 4.03 (m, 6H), 3.65 (dd, *J* = 5.7, 3.1 Hz, 2H), 3.59 (tdd, *J* = 5.2, 4.2, 1.3 Hz, 4H), 3.06 (dd, *J* = 5.7, 4.6 Hz, 2H), 2.57 (s, 2H).
